# Evidence Report on the Safety of Gastrointestinal Endoscopy in Patients on Glucagon-like Peptide-1 Receptor Agonists: A Systematic Review and Meta-Analysis

**DOI:** 10.3390/diagnostics15060770

**Published:** 2025-03-19

**Authors:** Zahid Ijaz Tarar, Umer Farooq, Ahtshamullah Chaudhry, Mustafa Gandhi, Abdallah El Alayli, Mark Ayoub, Baltej Singh, Ebubekir Daglilar, Nirav Thosani

**Affiliations:** 1 Department of Gastroenterology and Hepatology, School of Medicine, University of Missouri, Columbia, MO 65211, USA; mustafa.gandhi@health.missouri.edu; 2 Department of Gastroenterology and Hepatology, Saint Louis University, Saint Louis, MO 63103, USA; umer7513781@gmail.com; 3 Department of Medicine, St. Dominic Hospital, Jackson, MS 39216, USA; ahtsham_87@hotmail.com; 4 Department of Medicine, SSM Health, Saint Louis University, Saint Louis, MO 63103, USA; abdallahalayli@gmail.com; 5 Department of Medicine, Charleston Area Medical Center, Charleston, WV 25301, USA; 6 Department of Medicine, Christiana Care Union Hospital, Elkton, MD 21921, USA; aulakhmd@gmail.com; 7 Division of Gastroenterology and Hepatology, Charleston Area Medical Center, West Virginia University, Charleston, WV 25304, USA; ebubekirdaglilar@gmail.com; 8 Center for Interventional Gastroenterology at UTHealth (iGUT), McGovern Medical School at UTHealth, Houston, TX 77002, USA

**Keywords:** glucagon-like peptide receptor agonist, gastric residue, aspiration, diabetes mellitus, obesity

## Abstract

**Background/Objectives**: Glucagon-like peptide-1 receptor agonists are increasingly used worldwide for weight and hyperglycemia management. There is an ongoing debate on the presence of increased gastric residue, leading to complications such as aspiration and overall safety in patients receiving upper gastrointestinal endoscopy. We aimed to study the effect of GLP-RAs on endoscopy outcomes. **Methods**: We conducted a detailed search of online databases to select the studies which provided details of the effects of GLP-RAs on patients undergoing endoscopy. The outcomes of interest were odds of retained gastric content (RGC), aspiration risk, and aborted and repeated procedures. A random effect model was used to calculate the pooled odds of outcomes with a 95% CI. We further calculated the pooled odds of predictive factors associated with an increased rate of retained gastric residues in the study population. **Results**: We included 12 studies with a total of 105,515 patients, of which 32,144 were on GLP-1 RAs and 73,273 were in the control group. A total of 234 (0.73%) aspiration events in GLP-RA users were noted compared to 257 (0.35%) events in the control group. No increased odds (1.26, 95% CI 0.86–1.87, I^2^ 34%) of aspiration were found in GLP-1 users compared to the non-GLP-1 group. Patients on GLP-1 RA had increased RGC compared to the control group (OR 6.30, 95% CI 5.30–7.49, I^2^ 0%). The pooled odds of aborted (OR 5.50, 95% CI 3.25–9.32, I^2^ 0%) and repeated procedures (OR 2.19, 95% CI 1.42–3.38, I^2^ 0%) were significantly higher in GLP-1 RA users. Patients taking Tirazepatide had the highest percentage of RGC (18.9%), while exenatide users had the lowest rate (6.2%) of food retention. Patients undergoing concomitant colonoscopy were found to have significantly low pooled odds of RGC (OR 0.26, 95% CI 0.04–0.48). GLP-1 RAs use was independently associated with increased odds of RGC (3.91, 95% CI 3.21–4.62, I^2^ 0%). The results were homogenous and stayed consistent in the sensitivity analysis. **Conclusions**: Although the odds of RGC and aborted procedures are high in the GLP-1 RAs group compared to the control, no significant difference in the odds of aspiration was found between the two groups. Simple measures such as a clear liquid diet for 24 h, as routinely set for patients undergoing colonoscopy, may reduce the risk of retaining gastric residue in these patient populations.

## 1. Introduction

Glucagon-like peptide-1 receptor agonists (GLP1-RAs) are a class of medications primarily used for treating type 2 diabetes mellitus (T2DM). These medications affect glucose control by stimulating glucose-dependent insulin release from the pancreatic islets, leading to slowed down gastric emptying and reduced postprandial glucagon and appetite suppression [1]. The GLP-1RA-induced appetite suppression and decreased food intake is achieved by a combination of GLP-1 receptors’ activation in the periphery through vagal nerve stimulation and targeting GLP-1 receptors in the hypothalamus [2].

These medications lead to weight loss, a lower risk of hypoglycemia, and cardiovascular and renal health benefits [3]. GLP-1 RAs became available in 2005 for managing type 2 diabetes and obesity. There has been an astronomical increase in the use of these medications in recent years, with studies reporting an 11-fold increase in utilization from 2014 to 2022 [4,5]. Semaglutide, a drug in this class with a half-life of 7 days, has become widely popular owing to its convenient weekly dosing, favorable cardiometabolic profile, and weight loss benefits [6].

GLP-1 RA therapy predominantly causes gastrointestinal side effects such as nausea, vomiting, and diarrhea [7]. GLP-1-based medications have been linked to more serious gastrointestinal concerns, such as obstruction and symptomatic gastroparesis, when used for body weight reduction [8]. It has been proposed that measuring gastric emptying can be a surrogate for GLP-1 RA response and help assess whether a patient is suitable for long-term therapy [9]. An indirect method of gastric emptying is measuring the serum acetaminophen concentration after ingestion since it is only absorbed in the duodenum.

Gastroparesis increases the chances of residual gastric contents from delayed gastric emptying. Delayed gastric emptying has important anesthetic implications. Esophagogastroduodenoscopy (EGD) is a typically well-tolerated and safe method for screening, diagnosing, and treating gastrointestinal (GI) disorders. Thousands of endoscopic procedures involving the use of a native airway are conducted under anesthesia in the United States every day, where the risks of aspiration, procedure delays, inaccurate results, and overall healthcare costs are increased by the presence of gastric residue [10]. The American Society of Anesthesiologists advises delaying elective surgeries by one dose cycle with GLP-1RAs [11]. However, the American Gastroenterological Association (AGA) advises against ceasing medication the day before endoscopy in favor of instituting a liquid diet in asymptomatic patients and following conventional perioperative precautions [12]. The leading cause of this recommendation variation is the dearth of adverse event data in this patient demographic.

In recent times, comparative studies have investigated and attempted to quantify the risk caused by using GLP-1RA at the time of endoscopic procedures. We have performed a meta-analysis on these studies to study the data regarding the use of these drugs in the periprocedural setting to reduce the risk of adverse events.

## 2. Materials and Methods

**Figure 1:**
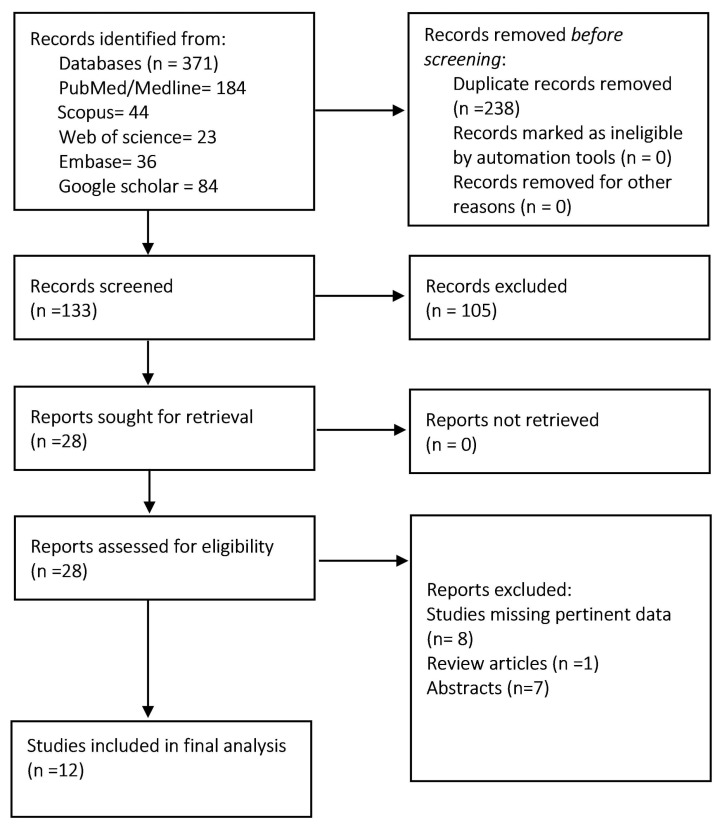
Prisma flow diagram of study selection.

We followed the meta-analysis of observational studies (MOOSE) guidelines and preferred reporting items for systematic review and meta-analysis to conduct this systematic review and meta-analysis [13,14] (Figure 1). The PRISMA statement checklist is provided in the Supplementary Materials.

### 2.1. Data Source and Search Strategy

We searched articles published in Web of Science, PubMed/Medline, Embase, and Scopus until August 2024. We also manually screened the references of the included studies. We used the following words to conduct the literature search: “Glucagon-like Peptide OR GLP-1 RA” AND “Gastric residue or gastric content OR retained food” AND “aspiration OR pneumonia” AND “Diabetes Mellitus” AND “Obesity”. We did not apply any limitations based on language, study design, or country of study. In addition to the free keywords, we also used the medical subject headings (MeSH) terms and Embase subject heading (EMTREE) terms. We further searched the bibliography of the selected articles to identify any missing articles. The literature search was conducted by two experienced investigators (ZIT and MG) and the University of Missouri Healthcare Science Library team.

### 2.2. Study Selection and Eligibility Criteria

We included all peer-reviewed studies that report data on RGC, aborted and repeated procedures, and aspiration in patients taking GLP-1 RAs. We excluded case reports, case series, and conference abstracts. We also excluded studies that reported data only on colonoscopy and GLP-1 RA use.

### 2.3. The PICO Criteria Were Used to Formulate the Inclusion Criteria

♦ Population: Patients above age 18 undergoing upper endoscopy (EGD) or concurrent EGD and colonoscopy.
♦ Intervention: Using GLP-1 RAs.
♦ Comparison: Not on GLP-1 RAs.
♦ Outcome: Risk of RGC and aspiration between these two groups.

### 2.4. Data Extraction

Two reviewers (ZIT and AC) extracted patient data and study characteristics. The information retrieved was the study’s type, design, year and duration, gastric residue, and aspiration rate. The patient information retrieved was age, gender, BMI, co-morbidities, indication for GLP-1 RA use, same-day colonoscopy, and insulin dependence.

### 2.5. Outcomes

We measured the pooled odds of aspiration, retained gastric content, and aborted and repeated procedures in the GLP-1 RAs group compared to the control. We further investigated the effect of DM, insulin dependence, obesity, age, and same-day colonoscopy on the rate of gastric residue. We also calculated the retained gastric residue rate in different GLP-1 RAs.

### 2.6. Data Synthesis and Statistical Analysis

We used the open meta-analysis software and Stata software 18 to conduct the analysis [15,16]. The random effect model within the Der Simonian–Laird method was used to calculate the pooled odds ratios and corresponding 95% CI. *p*-values <0.05 were considered significant. Sensitivity analysis was conducted by removing the individual studies from the results. Cochrane chi-square and I^2^ statistics assessed the variance and statistical heterogeneity. Heterogeneity was deemed significant if I^2^ was > 75%, moderate with I^2^ 50–75%, mild if I^2^ 25–50%, and minimal if I^2^ was less than 25%. Forest plots were generated to present the pooled outcomes. Funnel plots and rank order test for asymmetry were used to determine the publication bias.

### 2.7. Quality Assessment

We relied on the Methodological Index for Non-randomized Studies (MINORS) criteria to assess the quality of the included studies. We scored comparative studies on twelve items of MINORS criteria, and each item is scored from 0 to 2 (0 if not reported; 1 when reported but inadequate; and 2 when reported and adequate). Thus, a maximum ideal score of 24 could be obtained for comparative studies and 16 for non-comparative studies.

Based on MINORS criteria for non-randomized studies, the quality of the studies was classified as poor (score ≤5), fair (score 6–10), or high quality (≥11), as described previously [17]. As such, all the studies were rated as high quality. The quality assessment of studies is summarized in the Supplementary Table S1.

### 2.8. Certainty in Effect Estimates

The certainty of the evidence, also known as quality of the evidence or confidence in the effect estimate, was assessed using the Grading of Recommendations Assessments, Development, and Evaluation (GRADE) approach [18]. The final judgment can be high, moderate, low, or very low certainty. Evidence from non-randomized clinical trials starts as low certainty. However, it could be downgraded based on the risk of bias, imprecision, indirectness, inconsistency, and publication bias or upgraded based on confounders, significant effect, or dose-dependent effect. All evidence certainty assessment tables were created using the GRADEpro online tool [19] (Supplementary Table S2). 

## 3. Results

### 3.1. Search Results and Baseline Characteristics of the Study Population

The initial literature search identified 371 articles, of which 238 were excluded as duplicates. We reviewed the remaining 133 reports and finally shortlisted 28 studies relevant to our study question. After reading the full text of these articles and applying strict inclusion and exclusion criteria, the final selection of 12 articles was made (Figure 1). All the included studies were retrospective. A total of 105,417 patients were analyzed; 32,144 were on GLP-1 RAs and 73,273 were not on GLP-1 RAs. The mean age of the GLP-1 RAs group was 59.1, while that of the control group was 58.4. The mean body mass index (BMI) was 34.1 in the GLP-1 RA group compared to 31.6% in the control group. A total of 30,694 (95.4%) had diabetes in the GLP-1 RA group compared to 42,359 (57.8%) in the non-GLP-1 RA control group. The number of male patients in the GLP-1 RA group was 12,888 (40.1%) compared to 31,294 (42.7%) in the control group. Table 1 describes the study and patient characteristics.

### 3.2. Aspiration/Aspiration Pneumonia

**Figure 2:**
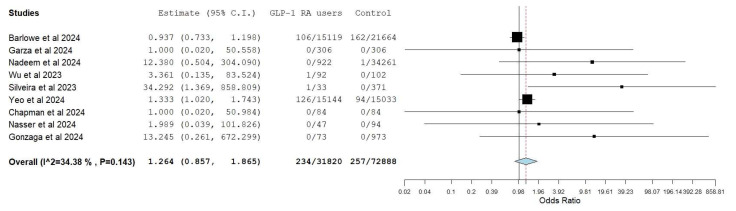
Random model pooled estimates of aspiration in GLP-1 RA users vs. control [20,21,23,26,27,28,29,30,31].

Nine studies [20,21,23,26,27,28,29,30,31] provided comparative data on aspiration or aspiration pneumonia in patients taking GLP-1 undergoing endoscopy. In total, 234 cases (0.73%) of aspiration were reported in 31,820 patients taking GLP-1 compared to 257 (0.35%) in 72,888 non-GLP-1 RA users. There was no statistical difference in the pooled odds (OR 1.26, 95% CI 0.86–1.87, I^2^ 34%) of aspiration in patients taking GLP-1 RA compared to the control group (Figure 2). The results remained consistent (OR 1.26 95% CI 0.86–1.87) after removing the individual studies from the results of the sensitivity analysis (Supplementary Figure S2a).

### 3.3. Retained Gastric Residue

**Figure 3:**
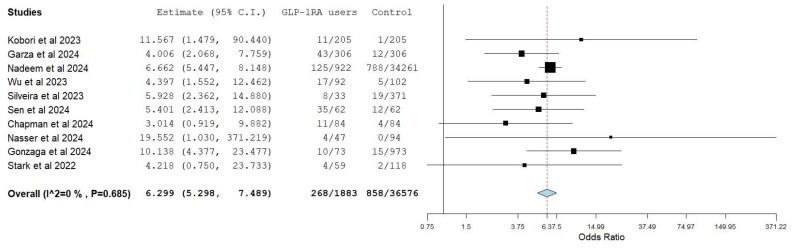
Random model pooled estimates of gastric residue in GLP-1 RA users vs. control [21,22,23,24,25,26,27,29,30,31].

Ten studies [21,22,23,24,25,26,27,29,30,31] analyzed and reported comparative data on gastric residue in patients taking GLP-1 and the control group. A total of 268 (14%) patients in the GLP-1 group had retained food compared to 858 (2.34%) patients in the control group. The patients taking GLP-1 had significantly higher pooled odds (OR 6.30, 95% CI 5.30–7.49, I^2^ 0%) of retained gastric content compared to the non-GLP-1 patient population (Figure 3). The results were same on leave one out sensitivity analysis (Supplementary Figure S2b). 

### 3.4. Aborted Procedures

**Figure 4:**
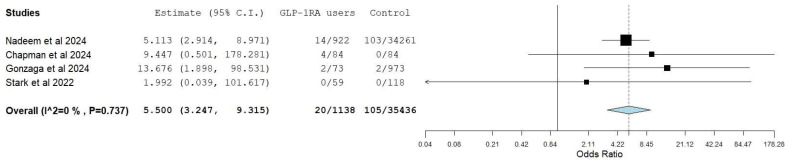
Random model pooled estimates of aborted procedures in GLP-1 RA users vs. control [23,25,29,30].

Four studies [23,25,29,30] reported the number of aborted endoscopy procedures in the GLP-1 RA and control groups. A total of 20 procedures (1.75%) were aborted in the GLP-1 group compared to 105 (0.3%) in the control group. The pooled odds (5.50, 90% CI 3.25–9.32, I^2^ 0%) of aborted procedures were significantly higher in the GLP-1 RA group (Figure 4). No change in the results was seen on the sensitivity analysis (Supplementary Figure S2c).

### 3.5. Repeated Procedures

In total, 22 (2.24%) patients in the GLP-1 RAs group required repeat endoscopic procedures compared to 377 (1.09%) patients in the control group, based on data provided by two studies [23,25], with pooled odds of 2.19 (95% CI 1.42–3.38, I^2^ 0%) (Supplementary Figure S1).

### 3.6. Predictors of Increased Gastric Residue

We further analyzed the predictive factors of RGC in GLP-1RA treatment group patients, as reported in Table 2. Male patients on GLP-1 had higher odds (OR 1.22, 95% CI 1.06–1.38, I^2^ 0%) of RGC than the female GLP-1 study population. An increase in BMI was not associated with higher odds of RGC (OR 1.05, 95% CI 0.98–1.11, I^2^ 90%) in patients taking GLP-1. Diabetic patients on GLP-1 had similar odds (OR 1.71, 95% CI 0.36–3.07) of RGC compared to non-diabetic patients taking GLP-1. Similarly, patients with higher HBA1C dependent on insulin had no added effect on food retention (OR 1.94, 95% 0.34–3.54, I^2^ 78%). Interestingly, GLP-1 RA group patients who underwent colonoscopy the same day had lower odds (OR 0.26, 95% CI 0.04–0.48) of food retention. After adjusting for covariates such as age, BMI, gender, DM, insulin use, and HBA1c, GLP-1 RA was independently associated with increased pooled odds (3.91, 95% CI 3.21–4.62, I^2^ 0%) of RGC.

### 3.7. Retained Gastric Residue in Different Types of GLP-1 RA

**Figure 5:**
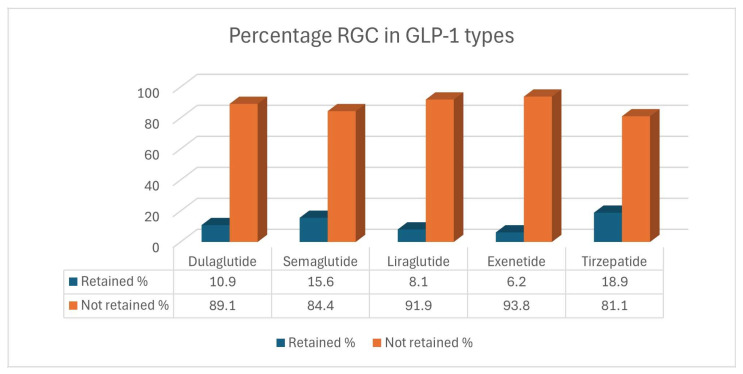
Percentage retained gastric residue in different types of GLP-1 RA.

We analyzed the rate of RGC for different kinds of GLP-1 agents. We found that patients taking Tirzepatide had the highest rate (18.9%) of retained gastric residue, followed by Semaglutide (15.6%), Dulaglutide (10.9%), Liraglutide (8.1%), and Exenatides (6.2%) (Figure 5).

### 3.8. Certainty of Evidence and Risk of Bias

The certainty in the evidence was found to range from very low to low for the reported outcomes. The certainty in the evidence started as low because the included studies are non-randomized trials. The certainty in the evidence was further downgraded due to concerns of imprecision and consistency between the included studies. The full GRADE assessment can be found in Supplementary Table S2. Funnel plots were created to assess publication bias. Though funnel plots are asymmetrical, the rank correlation test was negative for any publication bias (*p* = 0.61) (Supplementary Figure S3a,b).

## 4. Discussion

There has been a rapid increase in the use of glucagon-like peptide-1 receptor agonists (GLP-1 RA) in managing diabetes and weight loss. GLP-1 RA results in delayed gastric emptying, which can lead to increased retained gastric contents. An increased amount of food in the stomach is a well-known risk of aspiration during EGD. Even though they are extensively used, data on their safety in the perioperative period are limited [27,32,33]. GLP-1 plays a role in postprandial glucose maintenance via inhibiting glucagon- and glucose-dependent insulin secretion and slowing down gastric emptying; consequently, these actions lead to glycemic control and weight loss [34]. As they act on GLP-1 receptors in the stomach, they decrease gastric motility and emptying, raising concerns about increased retention of gastric content and risk of aspiration and decreasing mucosal visibility in patients undergoing endoscopic procedures [28].

In our meta-analysis, we investigated the effect of GLP-1 RAs on patients undergoing EGD and concurrent EGD and colonoscopy. We found that in the GLP-1 patient population, the odds of retained gastric content (OR 6.30, 95% CI 5.30–7.49, I^2^ 0%) are significantly higher, which further translated to an increased rate of aborted (5.50, 90% CI 3.25–9.32, I^2^ 0%) and repeated 2.19 (95% CI 1.42–3.38, I^2^ 0%) procedures. In total, 14% of the GLP-1 treatment group patients had retained gastric residue compared to 2.34% in the control group. A recent meta-analysis showed that GLP-1 use resulted in a 36 min delay in gastric emptying of solid content, but no delay was noted for liquids [35]. Our results reinforce these findings because we found that patients who received both EGD and colonoscopy concurrently had significantly lower odds (0.26, 95%CI 0.04–0.48) of retained gastric content, likely secondary to be on a clear liquid diet for colon preparation one day before the procedure. The American Gastroenterologist Association (AGA) does not recommend holding GLP-1 in asymptomatic patients; instead, it recommends a liquid diet before the day of the procedure [12]. In our analysis, we also found that the GLP-1RAs patient group has a higher rate (1.75%) of aborted procedures compared to 0.3% in the control group. A higher percentage of patients also required repeat endoscopy in the GLP-1RA group. Previous studies reported that a higher rate of gastric residue results in a higher number of aborted and terminated procedures, ranging from 2 to 30% [36,37]. However, these results should be interpreted carefully because only a limited number of studies have provided data on these outcomes, and the definition of retained gastric content was not uniform in different studies. Furthermore, holding GLP-1RA in all patients, especially if they are taking it for the management of diabetes, can lead to complications and poor disease control. Therefore, as recommended by the AGA, an individualized approach should be taken for these patients rather than stopping the GLP-1RAs for all.

Regardless of increased gastric content in the stomach, only 234 events (0.73%) of aspiration were reported in 31,280 patients in the GLP-1 treatment group compared to 257 (0.35%) events in the control group. There was no significant difference in the odds (OR 1.26, 95% CI 0.86–1.87, I^2^ 34%) of aspiration between both groups. The risk of aspiration during endoscopy is considered low generally. Previous studies by Anazco et al. [38] and Bohman et al. [39] showed a risk of aspiration in 4.8 cases per 10,000 and 4.6 cases per 10,000, respectively. Another retrospective study by Firkins et al. [40] found only 0.1% pulmonary aspiration risk in the GLP-1RA group. However, the results of our meta-analysis should be interpreted cautiously as studies have not provided independent factors associated with risk of aspiration in patients undergoing endoscopy, and evidence of certainty was very low. As stated earlier, holding GLP-1RA in every patient can lead to complications, so it seems reasonable to follow the AGA recommendations of an individualized approach and a standard perioperative eight-hour solid and two-hour liquid fast before endoscopy [12].

In addition to the above reported outcomes, we also calculated crude and adjusted odds of predictors resulting in increased gastric content. We found that GLP-1 RA use is independently associated with an increased risk of RGC (3.91, 95% CI 3.21–4.62, I^2^ 0%), which is in accordance with most of the studies included in our meta-analysis. The RGC significantly affects the gastric mucosal visibility and quality of the procedure, leading to procedure abortion. However, still, it is not seen to result in severe adverse events such as aspiration, endotracheal intubation, or mortality [29]. Therefore, the decision about stopping GLP-1RA before the endoscopic procedure should be well thought out, keeping in mind the adverse outcomes related to poor glycemic control in diabetic patients taking GLP-1RA. We did not find any significant difference in the odds of increased gastric content in patients with DM (OR 1.71, 95% CI 0.36–3.07, I^2^ 78%) and increase in BMI (OR 1.05, 95% CI 0.98–1.11, I^2^ 90%), though the results of previous studies are contrary. Garza et al. [21] did not show any difference in RGC, whereas Nadeem et al. [23] reported increased odds of RGC in patients with high BMI and DM. We found that Tirzepatide had the highest rate (18.9%) of retained gastric content compared to other GLP-1RA groups. This can be secondary to its dual mechanism of action affecting gastric inhibitory peptide and GLP-1 receptor activation [30,41].

One of the main strengths of this meta-analysis is the extensive nature of the study, with 105,417 patients. The larger sample size is more notable given the observed lower risk of aspiration. Second, it is the most updated analysis on this topic, including the highest number of fully published manuscripts. Third, we performed a detailed literature search and stringent inclusion and exclusion criteria to select the studies. Fourth, we measured the predictive pooled effect of patient characteristics, including BMI, gender, and diabetes, on the rate of gastric residue in patients taking GLP-1 RA. Fifth, we performed a sensitivity analysis to confirm the validity of our results. Furthermore, we provided the GRADE evidence of outcomes, which determine the strength of evidence and help in decision-making and establishing guidelines.

Though our analysis has many strong points, it still carries some limitations. First, the included studies are retrospective cohorts, with the risk of measured and unmeasured bias. Second, two studies were conducted on administrative databases, which can result in coding and documentation errors. Third, data on predictors of aspiration in GLP-1 RA users need to be improved. Fourth, more detailed data on the effect of different types of GLP-1 on gastric residue and aspiration risk need to be included.

## 5. Conclusions

In conclusion, our study showed that using GLP-1 RA results in the retention of gastric content, but that does not translate into an increased aspiration rate. Patients are taking GLP-1 for a variety of reasons and stopping it in every case can exacerbate their existing condition. Additionally, staying in the hospital for an extra day can significantly increase the financial burden on both the hospital and patients. Based on our analysis, we found no solid evidence for stopping GLP-1RA in all patients. It is reasonable to proceed with endoscopy following the standard perioperative fasting recommendation and liquid diet the day before the procedure.

## Figures and Tables

**Table 1:** Baseline Characteristics of the included studies.

Studies	Design, Year of Publication	Study Period	N	GLP-1 Users	Controls	Age (Years)		Male n (%)		BMI (kg/m^2^)		Diabetes Mellitus n (%)		Retained Gastric Content		Aspiration Events n (%)		Types of GLP-1 RA (n)	Gastric Residue Measurement
						GLP-1 Users	Control	GLP-1 User	Control	GLP-1 RA	Control	GLP-1 Users	Control	GLP-1 Users n (%)	Control n (%)	GLP-1 Users	Control		
Barlowe et al. [20]	Retrospective, 2024	2005–2021	36,783	15,119	21,664	55	57	6101 (40)	10,383 (48)	NR	NR	All study population had diabetes	-All study population had diabetes	NR	NR	106 (0.70%)	162 (0.75%)	NR	NR
Garza et al. [21]	Retrospective, 2024	2018–2023	612	306	306	61	62	147 (48)	154 (50)	32.9	32	269 (88)	268 (88)	43 (14)	12 (3)	0	0	Dulaglutide 108, SubQ Semaglutide 101,Oral Semaglutide 10, Liraglutide 49, Exenatide 13, Tirzepatide 12, Insulin-Lixisenatide combination 3, Insulin-Liraglutide combination 3	Any amount of solid content in the stomach
Kobori et al. [22]	Retrospective, 2023	2020–2022	410	205	205	70	72	163 (80)	154 (75)	NR	NR	NR	NR	11 (5.4)	1 (0.49)	NR	NR	Dulaglutide 85, Liraglutide 67, SubQ Semaglutide 29, Oral Semaglutide 7, Lixisenatide 5, Exenatide 1	Any amount of solid content in the stomach
Nadeem et al. [23]	Retrospective, 2024	2019–2023	35,183	922	34,261	57.1	53.9	364 (40)	14,070 (41)	36.4	30.5	756 (82)	5407 (15.8)	125 (13.6)	788 (2.3)	0	1 (0.002)	NR	NR
Sen et al. [24]	Prospective, 2024	2023	124	62	62	59	53	25 (40)	24 (39)	33.3	34.2	44 (71)	15 (24)	35 (56.5)	12 (19.4)	NR	NR	Semaglutide 39, Dulaglutide 14, Tirzepatide 9	
Stark et al. [25]	Retrospective, 2022	2015–2020	177	59	118	64	66	49 (83)	111 (94)	33	33	57 (97)	116 (98)	4 (6.8%)	2 (1.70)	NR	NR	Dulaglutide 33, Liraglutide 22, Exenatide 3, Semaglutide 1	Solid food in stomach
Wu et al. [26]	Retrospective, 2023	2019–2023	192	90	102	64.1	58.5	34 (38)	48 (47)	34	34	62 (69)	25 (25)	17 (18.9)	5 (4.9)	1 (1.1)	0	Semaglutide 70, Liraglutide 11, Dulaglutide 6, Tirzepatide 1, Combination 2	NR
Silveira et al. [27]	Retrospective, 2023	2021–2022	404	33	371	50.8	50.8	NR	NR	26.1	26.2	NR	NR	8 (24.2)	19 (5.1)	1 (3.0)	0	Semaglutide 33	Solid food in the stomach from esophagus to the pylorus. More than 0.8 mL/kg of liquid content.
Yeo et al. [28]	Retrospective, 2024	2018–2020	30,177	15,144	15,033	55.4	55.6	5941 (39)	5904 (39)	35.4	33.7	14,203 (94)	14,620 (97)	NR	NR	126 (0.8)	94 (0.6)	NR	NR
Chapman et al. [29]	Retrospective, 2024	2017–2023	168	84	84	53.9	54	24 (29)	26 (31)	40.7	31.2	73 (87)	71 (85)	11 (13.1)	4 (4.8)	0	0	Dulaglutide 41, Semaglutide 20, Liraglutide 14, Tirzepatide 7, Exenatide 2	POLPREP Score
Gonzaga et al. [30]	Retrospective, 2024	1/2023–6/2023	1046	73	973	57	55	21 (29)	350 (36)	34.4	27.7	53 (73)	131 (13.5)	10 (13.69)	15 (1.5)	0	0	Tirzepatide 11, Dulaglutide 22, Semaglutide 37, Exenatide 1, Liraglutide 1	Solid food visualized in the esophagus or stomach visualized during EGD.
Nasser et al. [31]	Retrospective, 2024	1/2023–6/2023	141	47	94	62.7	62.9	19 (40)	70 (74)	34.4	33.2	42 (89)	24 (26)	4 (8.5)	0	0	0	Semaglutide, dulaglutide, Tirzepatide, Liraglutide	Retained solid gastric content identified based on endoscopist reports

NR: not reported.

**Table 2:** Predictors of retained gastric content in GLP-1 users.

Predictors	Unadjusted Odds Ratio	95% CI	Heterogeneity I^2^ (%)	Adjusted Odds Ratios	95% CI with *p*-Value	Heterogeneity I^2^ (%)
GLP-1 RAs	4.72	2.92–6.51	55%	3.91	3.21–4.62	0%
Male Sex	0.87	0.47–1.28	0%	1.22	1.06–1.38	0%
Increasing BMI	1.04	0.96–1.11	73%	1.05	0.98–1.11	90.0%
Diabetes Mellitus	1.9	0.33–3.46	53%	1.94	0.34–3.54	78%
Insulin Dependence	NR	NR	0%	1.72	0.52–2.93	0%
Same Day Colonoscopy	0.74	0.10–1.39	92%	0.26	0.04–0.48	61%

## Data Availability

Data are available upon reasonable request.

## Supplementary Materials

The following supporting information can be downloaded at: https://www.mdpi.com/article/10.3390/diagnostics15060770/s1, File S1: PRISMA 2020 Checklist; File S2: Table S1: Quality assessment; Table S2: Grade evidence; Figure S1: Random model pooled estimates of Repeated procedures in GLP-1RA users vs. control; Figure S2a: Leave out one sensitivity analysis of aspiration risk in GLP-1RA users vs. Control; Figure S2b: Leave out one sensitivity analysis of RGC in GLP-1RA users vs. Control; Figure S2c: Leave out one sensitivity analysis of aborted procedures in GLP-1RA users vs. Control; Figure S3a: Funnel plot of aspiration risk; Figure S3b: Funnel plot of RGC.
