# Supplementary material for: Evidence Report on the Safety of Gastrointestinal Endoscopy in Patients on Glucagon-like Peptide-1 Receptor Agonists: A Systematic Review and Meta-Analysis
Source: Diagnostics (Basel). 2025 Mar 19;15(6):770. doi: 10.3390/diagnostics15060770 (PMC11941505; doi:10.3390/diagnostics15060770)
Supplement: Supplementary file 1 [file diagnostics-15-00770-s001.zip › diagnostics-3524968-supplementary File S2.pdf]

diagnostics

Table S1. Quality assessment.

Study	Clearly stated aim	Inclusion of consecutive patients	Prospective collection of data	Endpoints appropriate to the aim of the study	Unbiased assessment of the study endpoint	Follow-up period appropriate to the aim of the study	Loss to follow up less than 5%	Prospective calculation of the study size	An adequate control group	Contemporary groups	Baseline equivalence of groups	Adequate statistical analyses	Total Score
Barlowe et al.	2	2	2	2	0	2	2	2	1	2	1	2	20
Silveira et al.	2	2	2	2	0	2	0	1	2	2	1	2	18
Stark et al.	2	2	2	2	0	2	0	0	2	2	1	2	17
Wu et al.	2	2	2	2	0	2	0	2	2	2	1	2	19
Garza et al.	2	2	2	2	1	2	0	0	2	2	2	2	19
Kobori et al.	2	2	2	2	0	2	0	0	2	2	1	2	17
Nadeem et al	2	2	2	2	0	2	0	2	2	2	1	2	19
Sen at al.	2	2	2	2	0	2	0	2	2	2	1	2	19
Yeo et al.	2	2	2	2	0	2	0	2	2	2	1	2	19
Nasser et al.	2	2	2	2	0	2	0	2	2	2	1	2	19
Gonzaga et al.	2	2	2	2	0	2	0	2	2	2	1	2	19
Chapman et al.	2	2	2	2	1	2	0	2	2	2	1	2	20

Table S2. Grade evidence.

Summary of findings:

GLP-1 Users compared to Non- GLP-1 Users for Endoscopy

Patient or population: Endoscopy

Setting:

Intervention: GLP-1 Users

Comparison: Non- GLP-1 Users

Outcomes	Anticipated absolute effects* (95% CI)		Relative effect (95% CI)	№ of participants (studies)	Certainty of the evidence (GRADE)	Comments
	Risk with Non-GLP-1 Users	Risk with GLP-1 Users				
Retained gastric residue	23 per 1,000	**131 per 1,000** (113 to 152)	**OR 6.30** (5.30 to 7.49)	38457 (10 non-randomised studies)	⨁⨁◯◯ Low[a]	Serious indirectness and different study design.
Aspiration risk	4 per 1,000	**4 per 1,000** (3 to 7)	**OR 1.26** (0.86 to 1.86)	104708 (9 non-randomised studies)	⨁◯◯◯ Very low	The upper limit of the confidence interval suggests a positive effect, while the lower limit suggests minimal to no effect.
Aborted Procedure	3 per 1,000	**16 per 1,000** (10 to 27)	**OR 5.50** (3.25 to 9.32)	36574 (4 non-randomised studies)	⨁◯◯◯ Very low	Nonrandomized study design with high indirectness. Only 4 studies provided details on the outcome.
Retained gastric residue Gender	0 per 1,000	**0 per 1,000** (0 to 0)	**OR 1.19** (1.03 to 1.35)	(6 non-randomised studies)	⨁◯◯◯ Very low	The upper limit of the confidence interval suggests a small positive effect, while the lower limit suggests minimal to no effect.
Retained gastric residue (In Diabetes Mellitus patients)	0 per 1,000	**0 per 1,000** (0 to 0)	**OR 1.71** (0.36 to 3.07)	(6 non-randomised studies)	⨁◯◯◯ Very low	The upper limit of the confidence interval suggests a small positive effect, while the lower limit suggests minimal to no effect. High impercision.

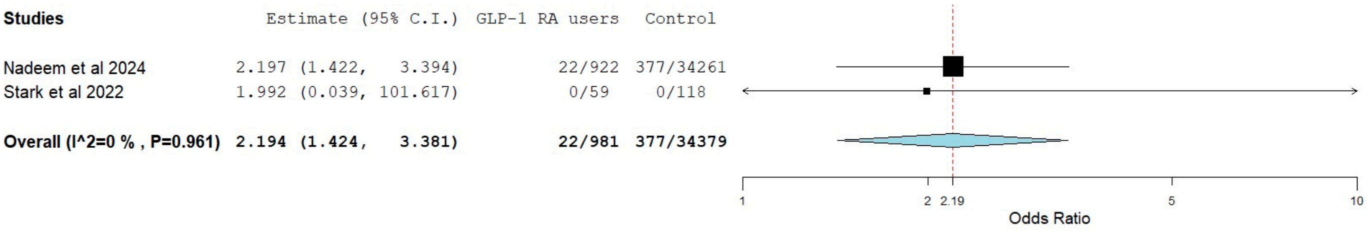

Figure S1. Random model pooled estimates of Repeated procedures in GLP-1RA users vs. control.

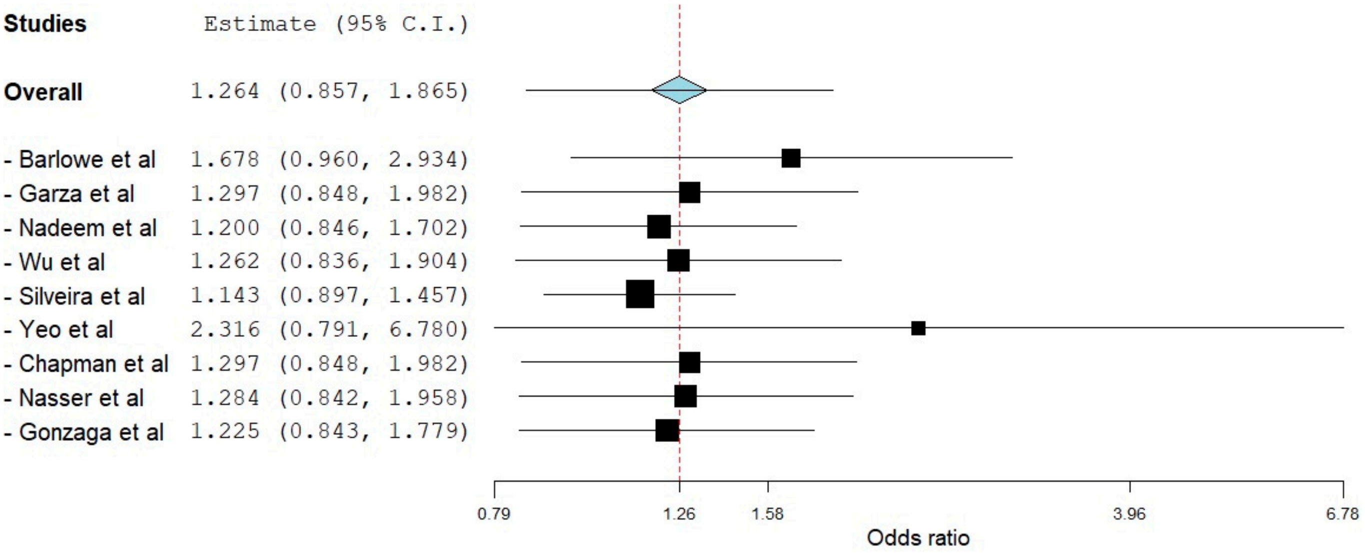

Figure S2a. Leave out one sensitivity analysis of aspiration risk in GLP-1RA users vs. Control.

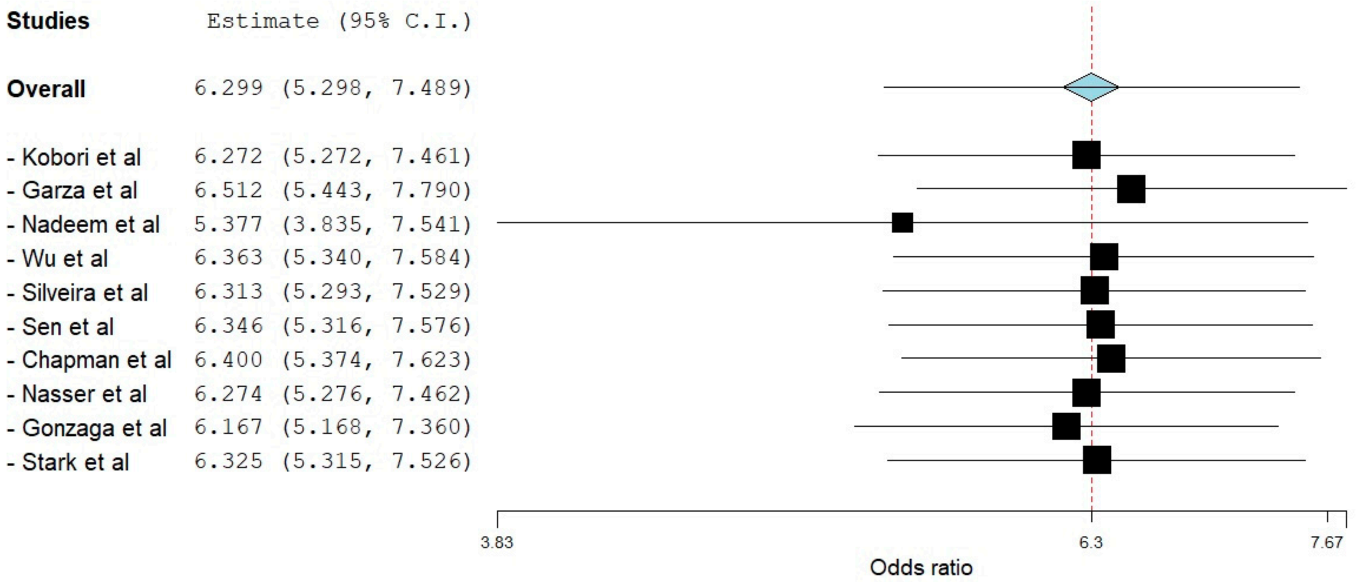

Figure S2b. Leave out one sensitivity analysis of RGC in GLP-1RA users vs. Control.

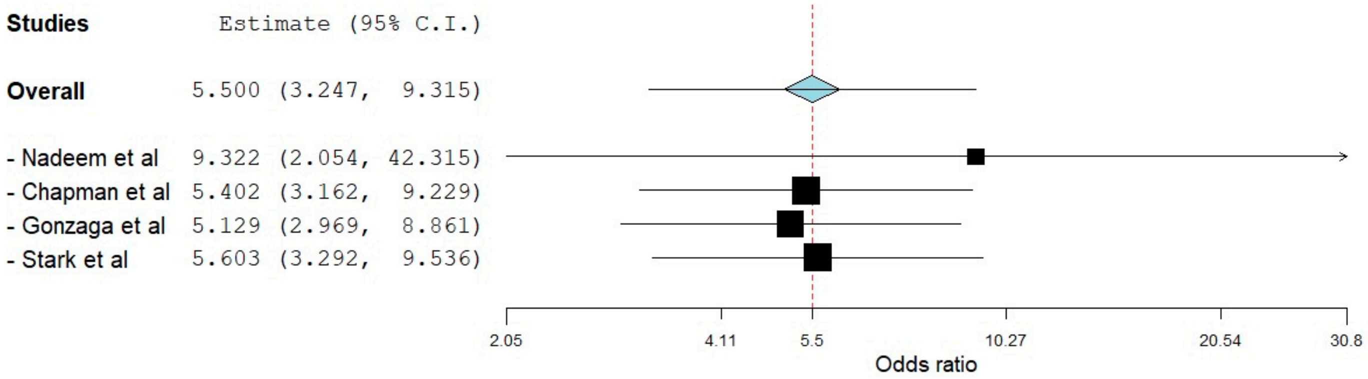

Figure S2c. Leave out one sensitivity analysis of aborted procedures in GLP-1RA users vs. Control.

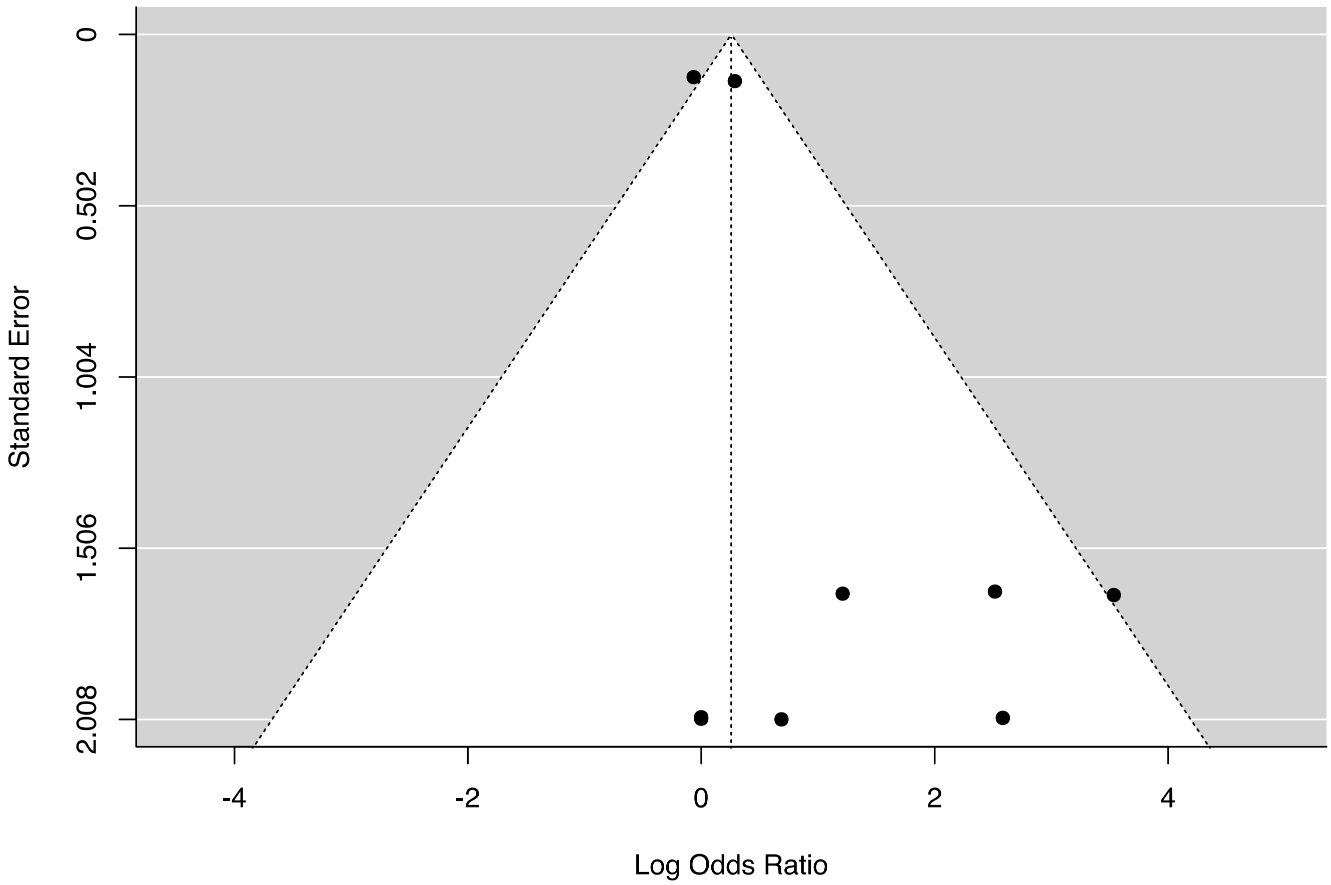

Figure S3a. Funnel plot of aspiration risk.

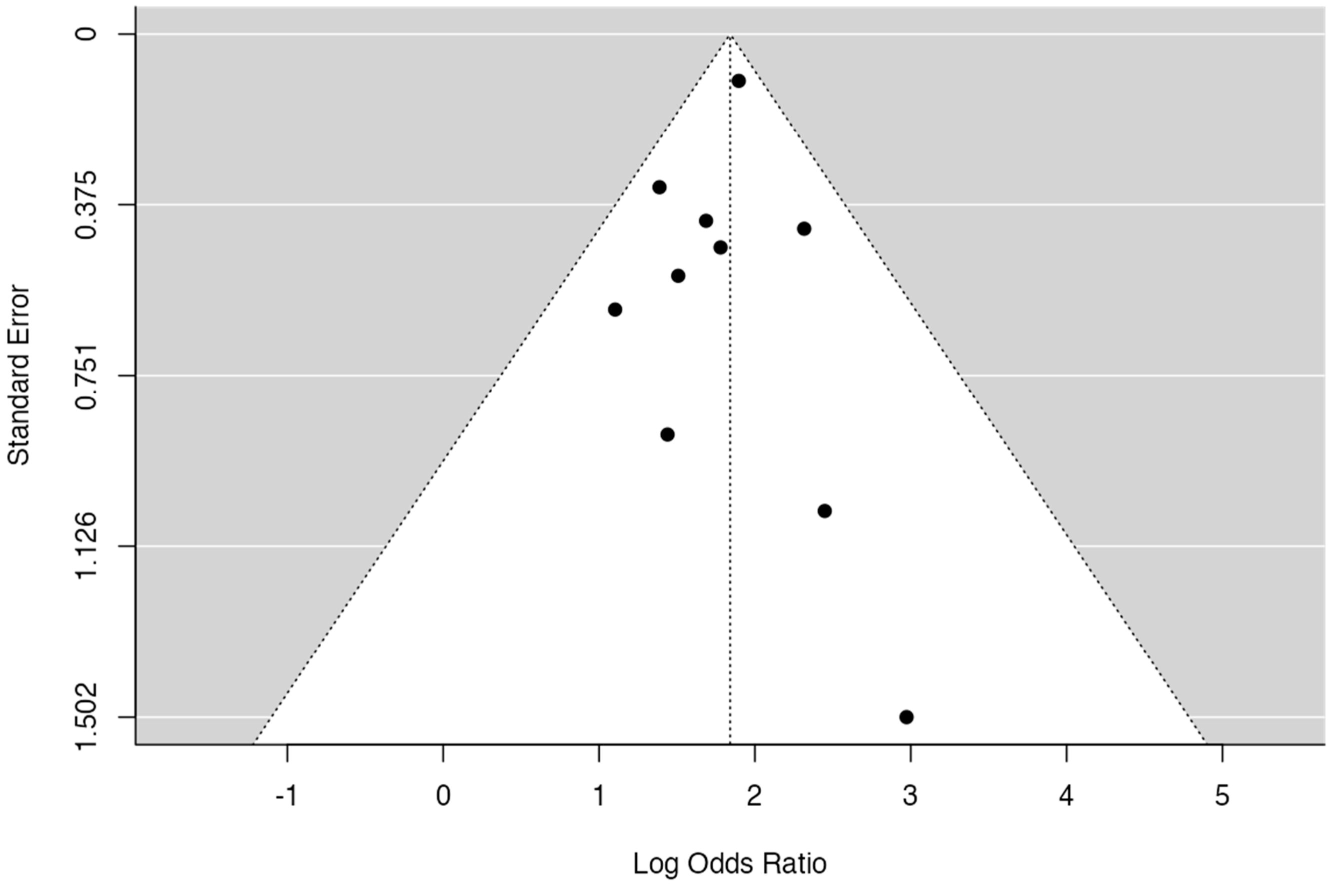

Figure S3b. Funnel plot of RGC.
